# The Modes of the Poisson Distribution of Order 3 and 4

**DOI:** 10.3390/e25040699

**Published:** 2023-04-21

**Authors:** Yeil Kwon, Andreas N. Philippou

**Affiliations:** 1 Department of Mathematics, University of Central Arkansas, Conway, AR 72035, USA; 2 Department of Mathematics, University of Patras, 26500 Patras, Greece; anphilip@math.upatras.gr

**Keywords:** poisson distribution of order *k*, discrete distribution, mode, most probable value, order 4

## Abstract

In this article, new properties of the Poisson distribution of order *k* with parameter λ are found. Based on them, the modes of the Poisson distributions of order k=3 and 4 are derived for λ in (0,1). They are 0, 3, 5, and 0, 4, 7, 8, respectively, for λ in specified subintervals of (0, 1). In addition, using Mathematica, computational results for the modes of the Poisson distributions of order k=2,3, and 4 are presented for λ in specified subintervals of (0,2).

## 1. Introduction

Following the papers of Philippou and Muwafi [1], Philippou et al. [2], Philippou [3,4,5], Aki et al. [6] and Aki [7], there has been an upsurge in the study of distributions of order *k* (distributions of runs) due to their theoretical importance and great applicability in reliability, start-up demonstration tests, sampling inspection, etc. See, e.g., Ling [8], Mohanty [9], Chang [10], Johnson et al. [11], Shmueli and Kohen [12], Balakrishnan and Koutras [13], Fu and Lou [14], Eryilmaz [15], Rakitzis and Antzoulakos [16], Dafnis et al. [17], Sengar et al. [18], Kwon [19], and references therein. However, the modes of these distributions are not yet known, except for the modes of the geometric distribution of order *k* and partial results for the mode(s) of the Poisson distribution of order *k* and the negative binomial distribution of the same order derived by Luo [20], Georghiou et al. [21], Philippou [22], Shao and Fu [23], and Georghiou et al. [24].

The Poisson distribution of order k(k≥1,integer) with parameter λ(>0) say $P_{k}\left(\lambda \right)$, has probability mass function (pmf)

(1) $$\begin{array}{c} f_{k}\left(x;\lambda \right)=e^{-k\lambda }\sum \frac{\lambda^{x_{1}+x_{2}+\cdots +x_{k}}}{x_{1}!x_{2}!\cdots x_{k}!},\;\;\;\;x=0,1,2,\ldots , \end{array}$$

where the summation is taken over all *k*-tuples of non-negative integers $x_{1},\;x_{2},\;\ldots ,x_{k}$ such that $x_{1}+2x_{2}+\cdots +kx_{k}=x.$

It was derived by Philippou et al. [2] as a limit of the negative binomial distribution of order *k*, and it was named so, since, for k=1, it reduces to the Poisson distribution with parameter λ. It is a special case, for $\lambda_{1}=\lambda_{2}=\cdots =\lambda_{k}=\lambda$, of the multiparameter Poisson distribution of order *k* (Philippou [25]), also known as *k* stuttering Poisson distribution (Galliher et al. [26], Patel [27]). The latter author discussed the estimation of the parameters of the triple and quadruple stuttering distributions and noted that the cases *k* = 2, 3, and 4 are more frequently observed in practice.

Let $m_{k,\lambda }$ denote the mode(s) of $f_{k}\left(x;\lambda \right)$, i.e., the value(s) of *x* for which $f_{k}\left(x;\lambda \right)$ attains its maximum. It is well known that $m_{1,\lambda }=\lambda$ or λ−1 if λ∈N and $m_{1,\lambda }=\left\lfloor \lambda \right\rfloor$, if λ does not belong to N, where ⌊α⌋ denotes the greatest integer part of α. Philippou [3] derived some properties of $f_{k}\left(x;\lambda \right)$ and posed the problem of finding its mode(s) for k≥2. Hirano et al. [28] presented several graphs of $f_{k}\left(x;\lambda \right)$ for λ∈(0,1) and 2≤k≤8, and Luo [20] derived the following lower bound inequality for $m_{k,\lambda }$,

$$\begin{array}{c} m_{k,\lambda }\geq k\lambda \sqrt[{k}]{k!}-\frac{1}{2}k\left(k+1\right),\;k\geq 1,\;\lambda >0. \end{array}$$

Georghiou et al. [21] employed the probability generating function of the Poisson distribution of order *k* to improve the lower bound of Luo [20] and also to give an upper bound for $m_{k,\lambda }$

(2) $$\begin{array}{c} \frac{1}{2}k\left(k+1\right)\left(\lambda -1\right)+1-\delta_{k,1}\leq m_{k,\lambda }\leq \left\lfloor \frac{1}{2}k\left(k+1\right)\lambda \right\rfloor =u_{k,\lambda },\;k\geq 1,\;\lambda >0, \end{array}$$

where $\delta_{k,1}$ denotes the Kronecker delta. With the bounds of $m_{k,\lambda }$ in (2), they showed that

(3) $$\begin{array}{c} m_{k,\lambda }=\frac{1}{2}k\left(k+1\right)\lambda -\left\lfloor \frac{k}{2}\right\rfloor ,\;2\leq k\leq 5,\;\lambda \in N. \end{array}$$

Using the upper bound $u_{k,\lambda }$ of (2) and the definition of $m_{k,\lambda }$, Philippou [22] found that:

(a) For any integer k≥1 and 0<λ<2/k(k+1), the Poisson distribution of order *k* has a unique mode $m_{k,\lambda }=0$.
(b) The Poisson distribution of order 2 has a unique mode $m_{k,\lambda }=0$ if $0<\lambda <\sqrt{3}-1$; it has two modes $m_{k,\lambda }=0$ and 2 if $0<\lambda =\sqrt{3}-1$, and it has a unique mode $m_{k,\lambda }=2$ if $\sqrt{3}-1<\lambda <1.$ (The number $\sqrt{3}-1$ is the positive root (say $r_{2}$) of the quadratic equation $\lambda^{2}+2\lambda -2=0$.)

**Remark** **1.** *Since the modes of the Poisson distribution of order k with parameter λ are defined as the values of x∈{0,1,2,…}, which maximize $f_{k}\left(x;\lambda \right)$, they are its most probable values and they may be obtained numerically for any given positive integer k and positive λ, from*

$$\begin{array}{c} f_{k}\left(m_{k,\lambda };\lambda \right)=\max\left\{f_{k}\left(x;\lambda \right)\;|\;\;x\in \left\{0,1,2,\ldots ,u_{k}\left(\lambda \right)\right\}\right\}. \end{array}$$

In the present short note, we derive some additional properties of $f_{k}\left(x;\lambda \right)$ and find the modes of the Poisson distribution of order k=3 and k=4 for 0<λ<1. Furthermore, Section 3 presents computational results for the modes of the Poisson distributions of order k=2,3, and 4 for λ∈ (0, 2). Finally, in Section 4, we briefly discuss our results, give the moment estimator of λ (>0) for k≥1, and indicate further research.

## 2. Main and Preliminary Results

The mode(s) of a discrete probability mass function is (are) its most probable value(s). In this section, we derive the modes of the Poisson distributions of order 3 and 4, respectively, when 0<λ<1 (see Propositions 1 and 2). In order to do so, we first state and prove three lemmas, regarding $h_{k}\left(x;\lambda \right)=e^{k\lambda }f_{k}\left(x;\lambda \right)$, which we use, along with relation (2), to prove the propositions.

Because of (1),

(4) $$\begin{array}{c} h_{k}\left(x;\lambda \right)=\sum \frac{\lambda^{x_{1}+x_{2}+\cdots +x_{k}}}{x_{1}!x_{2}!\cdots x_{k}!},\;x=0,1,2,\ldots ;\;\lambda >0, \end{array}$$

where the summation is taken over all *k*-tuples of non-negative integers $x_{1},\;x_{2},\;\ldots ,x_{k}$ such that $x_{1}+2x_{2}+\cdots +kx_{k}=x.$ Note that $h_{k}\left(0;\lambda \right)=1$ and $h_{k_{1}}\left(x;\lambda \right)=h_{k_{2}}\left(x;\lambda \right)$, for $1\leq x\leq k_{1}\leq k_{2}$.

Georghiou et al. [21] provided a recursive form of $f_{k}\left(x;\lambda \right)$ as

(5) $$\begin{array}{c} xf_{k}\left(x;\lambda \right)=\sum_{j=1}^{k}j\lambda f_{k}\left(x-j;\lambda \right),\;x\geq 1. \end{array}$$

It can be restated, in terms of $h_{k}\left(x;\lambda \right)$, as

(6) $$\begin{array}{c} xh_{k}\left(x;\lambda \right)=\left\{\begin{array}{cc} \sum_{j=1}^{x}j\lambda h_{k}\left(x-j;\lambda \right) & 1\leq x\leq k\\ \sum_{j=1}^{k}j\lambda h_{k}\left(x-j;\lambda \right) & x>k, \end{array}\right. \end{array}$$

with $h_{k}\left(0;\lambda \right)=1$.

**Lemma** **1.** *For 2≤x≤k and a fixed λ>0,*

$$\begin{array}{c} \lambda \leq h_{k}\left(x-1;\lambda \right)<h_{k}\left(x;\lambda \right). \end{array}$$

**Proof.**  To avoid the abuse of notation, let $h\left(x\right)\equiv h_{k}\left(x;\lambda \right)$. From (4), it is easy to see h(1)=λ. Using (6), for 2≤x≤k,

$$\begin{array}{cc} xh(x)-(x-1)h(x-1) & =\lambda \sum_{j=1}^{x}jh\left(x-j\right)-\lambda \sum_{j=1}^{x-1}jh\left(x-1-j\right)\\ & =\lambda \sum_{j=1}^{x}jh\left(x-j\right)-\lambda \sum_{j=2}^{x}\left(j-1\right)h\left(x-j\right)\\ & =\lambda \sum_{j=1}^{x}jh\left(x-j\right)-\lambda \sum_{j=2}^{x}jh\left(x-j\right)+\lambda \sum_{j=2}^{x}h\left(x-j\right)\\ & =\lambda h\left(x-1\right)+\lambda \sum_{j=2}^{x}jh\left(x-j\right)-\lambda \sum_{j=2}^{x}jh\left(x-j\right)+\lambda \sum_{j=2}^{x}h\left(x-j\right)\\ & =\lambda h\left(x-1\right)+\lambda \sum_{j=2}^{x}h\left(x-j\right)\\ & =\lambda \sum_{j=1}^{x}h\left(x-j\right) \end{array}$$

From (6), since $\left(x-1\right)h\left(x-1\right)=\sum_{j=1}^{x-1}j\lambda h\left(x-1-j\right)$, we have

$$\begin{array}{cc} x\left[h(x)-h(x-1)\right] & =xh(x)-(x-1)h(x-1)-h(x-1)\\ \; & =\lambda \left[\sum_{j=1}^{x}h\left(x-j\right)-\frac{1}{x-1}\sum_{j=1}^{x-1}jh\left(x-1-j\right)\right]\\ \; & =\lambda \left[\sum_{j=1}^{x}h\left(x-j\right)-\frac{1}{x-1}\sum_{j=2}^{x}\left(j-1\right)h\left(x-j\right)\right]\\ \; & =\lambda \left[h\left(x-1\right)+\sum_{j=2}^{x}h\left(x-j\right)-\frac{1}{x-1}\sum_{j=2}^{x}\left(j-1\right)h\left(x-j\right)\right]\\ \; & =\lambda \left[h\left(x-1\right)+\sum_{j=2}^{x}\left(1-\frac{j-1}{x-1}\right)h\left(x-j\right)\right]>0. \end{array}$$

Therefore, $h_{k}\left(x;\lambda \right)>h_{k}\left(x-1;\lambda \right)$ for 2≤x≤k with a fixed value of λ>0. □

**Lemma** **2.** *For k≥2 and 0<λ<1, the equation $h_{k}\left(k;\lambda \right)=h_{k}\left(0;\lambda \right)$ has exactly one root $\lambda =r_{k}$ $\left(0<r_{k}<1\right)$ such that*

$$\begin{array}{c} \left\{\begin{array}{cc} h_{k}\left(0;\lambda \right)>h_{k}\left(k;\lambda \right), & \mathrm{if}\;\;0<\lambda <r_{k}\\ h_{k}\left(0;\lambda \right)<h_{k}\left(k;\lambda \right), & \mathrm{if}\;\;r_{k}<\lambda <1. \end{array}\right. \end{array}$$

**Proof.**  First, note that $h_{k}\left(0;1\right)=1$ and $\lim_{\lambda \to 0^{+}}h_{k}\left(0;\lambda \right)=1$ because the relation (4) implies $h_{k}\left(0;\lambda \right)=1$ for λ>0. Since, for k≥1, $h_{k}\left(k;\lambda \right)$ is a polynomial function of λ with positive coefficient only and without constant term, we have $\lim_{\lambda \to 0^{+}}h_{k}\left(k;\lambda \right)=0$. Note that $h_{k}\left(1;\lambda \right)=\lambda$ and by Lemma 1, $h_{k}\left(1;\lambda \right)<h_{k}\left(2;\lambda \right)<\cdots <h_{k}\left(k;\lambda \right)$ for λ>0. Thus, $h_{k}\left(k;1\right)>h_{k}\left(1;1\right)=1$ for k≥2.

Second, let $g_{k}\left(\lambda \right)=h_{k}\left(k;\lambda \right)-h_{k}\left(0;\lambda \right)$. Then, since $\lim_{\lambda \to 0^{+}}g_{k}\left(\lambda \right)=\lim_{\lambda \to 0^{+}}h_{k}\left(k;\lambda \right)-\lim_{\lambda \to 0^{+}}h_{k}\left(0;\lambda \right)=0-1=-1<0,$ and $g_{k}\left(1\right)=h_{k}\left(k;1\right)-h_{k}\left(0;1\right)>h_{k}\left(1;1\right)-h_{k}\left(0;1\right)=1-1=0.$

Lastly, Since $h_{k}\left(x;\lambda \right)$ is a polynomial function with positive coefficients only, we have $\frac{\partial }{\partial \lambda }g_{k}\left(\lambda \right)>0$ for λ>0, which implies $g_{k}\left(\lambda \right)$ is an increasing function of λ for 0<λ<1.

Therefore, $g_{k}\left(\lambda \right)$ has a unique root, say $r_{k}$, between 0 and 1 with $h_{k}\left(0;\lambda \right)>h_{k}\left(k;\lambda \right)$, if $0<\lambda <r_{k}$ and $h_{k}\left(0;\lambda \right)<h_{k}\left(k;\lambda \right)$, if $r_{k}<\lambda <1$. □

**Lemma** **3.** *For k≥2 and $0<\lambda \leq r_{k}<1$,*

$$\begin{array}{c} h_{k}\left(k;\lambda \right)>h_{k}\left(k+1;\lambda \right), \end{array}$$

*where $r_{k}$ is defined in Lemma 2.*

**Proof.**  For notational simplicity, let $h\left(x\right)\equiv h_{k}\left(x;\lambda \right)$. From (6), we have

$$\begin{array}{c} kh\left(k\right)=\sum_{j=1}^{k}j\lambda h_{k}\left(k-j\right)\;\;\;\;\;\mathrm{and}\;\;\;\;\;\left(k+1\right)h_{k}\left(k+1\right)=\sum_{j=1}^{k}j\lambda h_{k}\left(k+1-j\right). \end{array}$$

Thus, with h(0)=1,

(7) $$\begin{array}{cc} k\left[h(k)-h(k+1)\right]\; & =\sum_{j=1}^{k}j\lambda h\left(k-j\right)-\sum_{j=1}^{k}j\lambda h\left(k+1-j\right)+h\left(k+1\right)\\ \; & =\sum_{j=1}^{k}j\lambda h\left(k-j\right)-\sum_{j=0}^{k-1}\left(j+1\right)\lambda h\left(k-j\right)+h\left(k+1\right)\\ \; & =\sum_{j=1}^{k}j\lambda h\left(k-j\right)-\sum_{j=0}^{k-1}j\lambda h\left(k-j\right)-\sum_{j=0}^{k-1}\lambda h\left(k-j\right)+h\left(k+1\right)\\ \; & =k\lambda h\left(0\right)-\sum_{j=0}^{k-1}\lambda h\left(k-j\right)+h\left(k+1\right)\\ \; & =\lambda \left(k-\sum_{j=0}^{k-1}h\left(k-j\right)\right)+h\left(k+1\right). \end{array}$$

Since each $h_{k}\left(1;\lambda \right),\;\;h_{k}\left(2;\lambda \right)\;,\ldots ,\;\;h_{k}\left(k;\lambda \right)$ are increasing functions of lambda, they all have the maximum value at $\lambda =r_{k}$ on $\lambda \in (0,r_{k}]$. Moreover, by Lemma 1 and the definition of $r_{k}$, we have $h_{k}\left(1;r_{k}\right)<h_{k}\left(2;r_{k}\right)<\cdots <h_{k}\left(k;r_{k}\right)=1.$ Therefore, for $0<\lambda \leq r_{k}$, the first term of (7) can be written as

$$\begin{array}{cc} k-\sum_{j=0}^{k-1}h_{k}\left(k-j;\lambda \right) & \geq k-\sum_{j=0}^{k-1}h_{k}\left(k-j;r_{k}\right)\\ \; & =k-\left[h_{k}\left(k;r_{k}\right)+h_{k}\left(k-1;r_{k}\right)+\cdots +h_{k}\left(1;r_{k}\right)\right]\\ \; & >k-k\left[(h_{k}\left(k;r_{k}\right)\right]=0, \end{array}$$

and it implies $h_{k}\left(k;\lambda \right)>h_{k}\left(k+1;\lambda \right).$ □

**Proposition** **1.** *Let $m_{3,\lambda }$ denote the mode(s) of the Poisson distribution of order 3 with parameter λ(>0). Let $r_{3}$ (=0.6016791318…) and $r_{3,5}$ (=0.9962030611…) be the positive roots of the equations $\lambda^{3}+6\lambda^{2}+6\lambda -6=0$, and $\lambda^{4}+20\lambda^{3}+100\lambda^{2}-120=0$, respectively. Then*

$$\begin{array}{c} m_{3,\lambda }=\left\{\begin{array}{cc} 0, & \;\mathrm{if}\;\;\;0<\lambda <r_{3},\\ 0\;\mathrm{and}\;3, & \;\mathrm{if}\;\;\;\lambda =r_{3},\\ 3, & \;\mathrm{if}\;\;\;r_{3}<\lambda <r_{3,5},\\ 3\;\mathrm{and}\;5, & \;\mathrm{if}\;\;\;\lambda =r_{3,5},\\ 5, & \;\mathrm{if}\;\;\;r_{3,5}<\lambda <1. \end{array}\right. \end{array}$$

**Proof.**  The Equation (2) implies

$$0\leq m_{3,\lambda }\leq \left\lfloor \frac{3}{2}\left(3+1\right)\lambda \right\rfloor =\left\lfloor 6\lambda \right\rfloor \leq 5,\;\mathrm{for}\;0<\lambda <1.$$

Hence, it is enough we compare the magnitudes of $f_{3}\left(x;\lambda \right)$, or the magnitudes of $h_{3}\left(x;\lambda \right)$ for 0≤x≤5. By Lemma 1, we have $h_{3}\left(1;\lambda \right)<h_{3}\left(2;\lambda \right)<h_{3}\left(3;\lambda \right)$ for 0<λ<1, and, by Lemma 2 and 3, it is given $1=h_{3}\left(0;\lambda \right)>h_{3}\left(3;\lambda \right)>h_{3}\left(4;\lambda \right)$ for $0<\lambda <r_{3}$. In addition, the maximum of $h_{3}\left(5;\lambda \right)$ on $\lambda \in (0,r_{3}]$ is $h_{3}\left(5;r_{k}\right)=r_{3}^{2}+r_{3}^{3}+\frac{1}{6}r_{3}^{4}+\frac{1}{120}r_{3}^{5}<1$. Hence, $m_{3,\lambda }=0$ for $0<\lambda <r_{3}$.

Furthermore, $h_{3}\left(3;\lambda \right)>h_{3}\left(4;\lambda \right)$ for 0<λ<1. To see this, let $d_{k}\left(x;\lambda \right)=h_{k}\left(k;\lambda \right)-h_{k}\left(x;\lambda \right)$. Then

$$\begin{array}{cc} d_{3}\left(4;\lambda \right) & =h_{3}\left(3;\lambda \right)-h_{3}\left(4;\lambda \right)\\ & =\left(\lambda +\lambda^{2}+\frac{1}{6}\lambda^{3}\right)-\left(\frac{3}{2}\lambda^{2}+\frac{1}{2}\lambda^{3}+\frac{1}{24}\lambda^{4}\right)\\ \; & =\lambda -\frac{1}{2}\lambda^{2}-\frac{1}{3}\lambda^{3}-\frac{1}{24}\lambda^{4}, \end{array}$$

with $\frac{\partial^{2}}{\partial \lambda^{2}}d_{3}\left(4;\lambda \right)=-1-2\lambda -\lambda^{2}/2<0,$ which implies that $d_{3}\left(4;\lambda \right)$ is concave down for λ>0. Since $\lim_{\lambda \to 0^{+}}d_{3}\left(4;\lambda \right)=0^{+}$, and $d_{3}\left(4;1\right)=1/8>0$, we have $h_{3}\left(3;\lambda \right)>h_{3}\left(4;\lambda \right)$ for 0<λ<1. Hence, it suffices that we compare the magnitude of $h_{3}\left(0;\lambda \right)$, $h_{3}\left(3;\lambda \right)$ and $h_{3}\left(5;\lambda \right)$ to find the modes for $r_{3}<\lambda <1$.

$$\begin{array}{cc} d_{3}\left(5;\lambda \right) & =h_{3}\left(3;\lambda \right)-h_{3}\left(5;\lambda \right)\\ \; & =\left(\lambda +\lambda^{2}+\frac{1}{6}\lambda^{3}\right)-\left(\lambda^{2}+\lambda^{3}+\frac{1}{6}\lambda^{4}+\frac{1}{120}\lambda^{5}\right)\\ & =\lambda -\frac{5}{6}\lambda^{3}-\frac{1}{6}\lambda^{4}-\frac{1}{120}\lambda^{5}. \end{array}$$

For λ>0, $d_{3}\left(5;\lambda \right)$ is concave down because $\frac{\partial^{2}}{\partial \lambda^{2}}d_{3}\left(5;\lambda \right)=-5\lambda -2\lambda^{2}-\lambda^{3}/6<0$. Since $\lim_{\lambda \to 0^{+}}d_{3}\left(5;\lambda \right)=0^{+}$ and $d_{3}\left(5;1\right)=-1/120$, the equation $d_{3}\left(5;\lambda \right)=0$, equivalently $\lambda^{4}+20\lambda^{3}+100\lambda^{2}-120=0,$ has one positive root, say $r_{3,5}$. Thus, $h_{3}\left(3;\lambda \right)>h_{3}\left(5;\lambda \right)$ for $0<\lambda <r_{3,5}$ and $h_{3}\left(3;\lambda \right)<h_{3}\left(5;\lambda \right)$ for $r_{3,5}<\lambda <1$. Since $0<r_{3}=0.601679$…$<r_{3,5}=0.996203$…<1, we have

$$\begin{array}{c} \left\{\begin{array}{cc} f_{3}\left(0;\lambda \right)>f_{3}\left(3;\lambda \right)>f_{3}\left(5;\lambda \right), & \;\mathrm{if}\;\;\;0<\lambda <r_{3},\\ f_{3}\left(3;\lambda \right)>f_{3}\left(0;\lambda \right)\;\;\;\;\&\;\;\;\;f_{3}\left(3;\lambda \right)>f_{3}\left(5;\lambda \right), & \;\mathrm{if}\;\;\;r_{3}<\lambda <r_{3,5},\\ f_{3}\left(5;\lambda \right)>f_{3}\left(3;\lambda \right)>f_{3}\left(0;\lambda \right), & \;\mathrm{if}\;\;\;r_{3,5}<\lambda <1. \end{array}\right. \end{array}$$

□

**Proposition** **2.** *Let $m_{4,\lambda }$ denote the mode(s) of the Poisson distribution of order 4 with parameter λ(>0), and let $r_{4}$ (=0.5203510176…), $r_{4,7}$ (=0.7947408725…), and $r_{7,8}$ (=0.8944652714…), respectively, be the positive roots of the equations $\lambda^{4}+12\lambda^{3}+36\lambda^{2}+24\lambda -24=0$, $\lambda^{6}+42\lambda^{5}+630\lambda^{4}+3990\lambda^{3}+7560\lambda^{2}-2520\lambda -5040=0$, and $\lambda^{6}+48\lambda^{5}+840\lambda^{4}+6720\lambda^{3}+18,480\lambda^{2}-20,160=0$. Then*

$$\begin{array}{c} m_{4,\lambda }=\left\{\begin{array}{cc} 0, & \;\mathrm{if}\;\;\;0<\lambda <r_{4},\\ 0\;\mathrm{and}\;4, & \;\mathrm{if}\;\;\;\lambda =r_{4},\\ 4, & \;\mathrm{if}\;\;\;r_{4}<\lambda <r_{4,7},\\ 4\;\mathrm{and}\;7, & \;\mathrm{if}\;\;\;\lambda =r_{4,7},\\ 7, & \;\mathrm{if}\;\;\;r_{4,7}<\lambda <r_{7,8},\\ 7\;\mathrm{and}\;8, & \;\mathrm{if}\;\;\;\lambda =r_{7,8},\\ 8, & \;\mathrm{if}\;\;\;r_{7,8}<\lambda <1. \end{array}\right. \end{array}$$

**Proof.**  The Equation (2) implies

$$0\leq m_{4,\lambda }\leq \left\lfloor \frac{4}{2}\left(4+1\right)\lambda \right\rfloor =\left\lfloor 10\lambda \right\rfloor \leq 9,\;\mathrm{for}\;0<\lambda <1.$$

Hence, it is enough we compare the magnitudes of $f_{4}\left(x;\lambda \right)$, or the magnitudes of $h_{4}\left(x;\lambda \right)$ for 0≤x≤9. By Lemma 1, we have $h_{4}\left(1;\lambda \right)<h_{4}\left(2;\lambda \right)<h_{4}\left(3;\lambda \right)<h_{4}\left(4;\lambda \right)$. Thus, we will compare the magnitudes of $h_{4}\left(x;\lambda \right)$ for 4≤x≤9, and $h_{4}\left(0;\lambda \right).$ They are given by

$$\begin{array}{cc} h_{4}\left(4;\lambda \right) & =\lambda +\frac{3}{2}\lambda^{2}+\frac{1}{2}\lambda^{3}+\frac{1}{24}\lambda^{4}\\ h_{4}\left(5;\lambda \right) & =2\lambda^{2}+\lambda^{3}+\frac{1}{6}\lambda^{4}+\frac{1}{120}\lambda^{5}\\ h_{4}\left(6;\lambda \right) & =\frac{3}{2}\lambda^{2}+\frac{5}{3}\lambda^{3}+\frac{5}{12}\lambda^{4}+\frac{1}{24}\lambda^{5}+\frac{1}{720}\lambda^{6}\\ h_{4}\left(7;\lambda \right) & =\lambda^{2}+2\lambda^{3}+\frac{5}{6}\lambda^{4}+\frac{1}{8}\lambda^{5}+\frac{1}{120}\lambda^{6}+\frac{1}{5040}\lambda^{7}\\ h_{4}\left(8;\lambda \right) & =\frac{1}{2}\lambda^{2}+2\lambda^{3}+\frac{31}{24}\lambda^{4}+\frac{7}{24}\lambda^{5}+\frac{7}{240}\lambda^{6}+\frac{1}{720}\lambda^{7}+\frac{1}{40,320}\lambda^{8}\\ h_{4}\left(9;\lambda \right) & =\frac{5}{3}\lambda^{3}+\frac{5}{3}\lambda^{4}+\frac{13}{24}\lambda^{5}+\frac{7}{90}\lambda^{6}+\frac{1}{180}\lambda^{7}+\frac{1}{5040}\lambda^{8}+\frac{1}{362,880}\lambda^{9} \end{array}$$

Note that $h_{4}\left(x;\lambda \right)$, for 4≤x≤9, are strictly increasing functions of λ and $h_{4}\left(x;0\right)$ = 1. Table 1 displays the function values of $h_{4}\left(x;\lambda \right)$ for 4≤x≤9 with λ=0.5,0.6,0.7,0.8,0.9,1.0, and 1.1. From the function values of the Table 1, we can see $h_{4}\left(4;\lambda \right)$ = 1 has a root $r_{4}\in \left(0.5,0.6\right)$, $h_{4}\left(4;\lambda \right)$ = $h_{4}\left(7;\lambda \right)$ has a root $r_{4,7}\in \left(0.7,0.8\right)$, and $h_{4}\left(7;\lambda \right)$ = $h_{4}\left(8;\lambda \right)$ has a root $r_{7,8}\in \left(0.8,0.9\right).$

Therefore,

$$\begin{array}{c} \left\{\begin{array}{cc} f_{4}\left(0;\lambda \right)>f_{4}\left(x;\lambda \right)\;\mathrm{where}\;1\leq x\leq 9, & \;\mathrm{if}\;\;\;0<\lambda <r_{4},\\ f_{4}\left(4;\lambda \right)>f_{4}\left(x;\lambda \right)\;\mathrm{where}\;0\leq x\leq 3,\;\mathrm{or}\;5\leq x\leq 9 & \;\mathrm{if}\;\;\;r_{4}<\lambda <r_{4,7},\\ f_{4}\left(7;\lambda \right)>f_{4}\left(x;\lambda \right)\;\mathrm{where}\;0\leq x\leq 6,\;\mathrm{or}\;8\leq x\leq 9 & \;\mathrm{if}\;\;\;r_{4,7}<\lambda <r_{7,8},\\ f_{4}\left(8;\lambda \right)>f_{4}\left(x;\lambda \right)\;\mathrm{where}\;0\leq x\leq 7,\;\mathrm{or}\;x=9 & \;\mathrm{if}\;\;\;r_{7,8}<\lambda <1. \end{array}\right. \end{array}$$

□

## 3. More Computational Resutls

This section provides more computational results using the computer algebra system Mathematica. Table 2 shows the modes of Poisson distribution of order k=2,3, and 4 for 0<λ<2. For λ>1, we observe that the mode values frequently change as λ increases. However, for every value of *k*, the first two modes are 0 and *k* for some subintervals of λ between zero and one. We also note that for k=2,3, and 4, $m_{k,1}=2,\;5,\;$ and 8, (the modes of the Poisson distribution of order *k* with λ=1) as it should, in accordance with (3).

## 4. Moment Estimation of the Parameter λ of $P_{k}\left(\lambda \right)$, Discussion, and Further Research

Despite the upsurge of the study of distributions of order *k* or runs since the early 1980s, their modes, due to the difficulty of obtaining them, are not known, except for the mode of the geometric distribution of order *k* and partial results for the modes of the negative binomial and Poisson distributions of order *k*. Their probability generating functions, however, and moments are well known.

The mean and variance of $P_{k}\left(\lambda \right)$, for example, are (a) k(k+1)λ/2 and (b) k(k+1)(2k+1)λ/6 (see, e.g., Philippou [3,25]). By means of them and the method of moments estimation, we now give the moment estimator $\hat{\lambda }$ of λ of $P_{k}\left(\lambda \right)$. Let $X_{1},X_{2},\ldots ,X_{n}$ be a random sample of size *n* from $P_{k}\left(\lambda \right)$, and set $\bar{X}=\left(X_{1}+X_{2}+\cdots +X_{n}\right)/n$. Then, the moment estimator of λ is $\hat{\lambda }=2\bar{X}/\left[k\left(k+1\right)\right]$. It is unbiased, and has variance $Var\left(\hat{\lambda }\right)=2\left(2k+1\right)\lambda /\left[3k\left(k+1\right)n\right]$. In fact, by the method of moments and (a), $\bar{X}=k\left(k+1\right)\hat{\lambda }/2$, which implies $\hat{\lambda }=2\bar{X}/\left[k\left(k+1\right)\right]$. It follows by (a) and (b), respectively, that $\hat{\lambda }$ is unbiased for λ, since $E\left(\hat{\lambda }\right)=2E\left(\bar{X}\right)/\left[k\left(k+1\right)\right]=\lambda$, and has variance $Var\left(\hat{\lambda }\right)=4Var\left(\bar{X}\right)/\left[k^{2}{\left(k+1\right)}^{2}\right]=\left[4/k^{2}{\left(k+1\right)}^{2}\right]\cdot \left[k\left(k+1\right)\left(2k+1\right)\lambda /\left(6n\right)\right]=2\left(2k+1\right)\lambda /\left[3k\left(k+1\right)n\right]$, which was to be shown.

In the present article, in addition to the above paragraph regarding $P_{k}\left(\lambda \right)$, we derived a few new properties of the Poisson distribution of order *k*, and using them, along with a result of Georghiou et al. [21], we found the modes or most probable values of the Poisson distributions of order 3 and 4 for λ in the interval (0,1). In addition, using Mathematica and a personal computer, we found the modes of the Poisson distributions of order 2, 3, and 4 for λ∈(0,2). We observe that for *k* = 2, 3, and 4, the first two modes are 0 for $0<\lambda <r_{k}$, and *k* for $r_{k}<\lambda <r_{k}+l_{k}$, where $l_{k}$ stands for the length of the interval on which *k* is the mode of the Poisson distribution of order *k*. Further research may include several interesting problems: Is it generally true that $m_{k,\lambda }=0$ for k≥2 and $0<\lambda <r_{k}$, and $m_{k,\lambda }=k$ for k≥2 and $r_{k}<\lambda <r_{k}+l_{k}$? Does $r_{k}$ decrease as *k* increases? If it does, how fast is $r_{k}$ decreasing? What positive integers cannot be modes of the Poisson distribution of order *k*?

## Figures and Tables

**Table 1:** The function values of $h_{4}\left(x;\lambda \right)$ for x=0 and 4≤x≤10 with λ=0.5, 0.6, 0.7, 0.8, 0.9, 1.0, and 1.1. The bolded value in each column stands for the maximum of $h_{4}\left(x;\lambda \right)$, which implies $m_{4,\lambda }=x\;$ with a value of λ given in the corresponding column. Note that $0.5<r_{4}<0.6<0.7<r_{4,7}<0.8<r_{7,8}<0.9.$ The function values are calculated based on substantially tight grid for λ. The table displays only the meaningful λ values.

λ							
*x*	0.5	0.6	0.7	0.8	0.9	1.0	1.1
0	**1.0000**	1.0000	1.0000	1.0000	1.0000	1.0000	1.0000
4	0.9401	**1.2534**	**1.6165**	2.0331	2.5068	3.0417	3.6415
5	0.6357	0.9582	1.3644	1.8630	2.4633	3.1750	4.0084
6	0.6107	0.9573	1.4139	1.9980	2.7287	3.6264	4.7129
7	0.5561	0.9101	1.3981	**2.0485**	2.8931	3.9669	5.3085
8	0.4653	0.8035	1.2937	1.9766	**2.8989**	**4.1139**	5.6823
9	0.3307	0.6219	1.0725	1.7351	2.6724	3.9585	5.6799
10	0.2686	0.5273	0.9457	1.5861	2.5258	3.8593	**5.7004**

**Table 2:** The modes of Poisson distribution of order k=2,3, and 4 for 0<λ<2. The lower and upper bounds of λ are the approximated values.

k=2			k=3			k=4		
Mode	Interval of λ		Mode	Interval of λ		Mode	Interval of λ	
0	(0.0000,0.7321)		0	(0.0000,0.6017)		0	(0.0000,0.5204)	
2	(0.7321,1.3412)		3	(0.6017,0.9962)		4	(0.5204,0.7947)	
4	(1.3412,1.8851)		5	(0.9962,1.0612)		7	(0.7947,0.8945)	
5	(1.8851,2.0000)		6	(1.0612,1.3881)		8	(0.8945,1.0950)	
			7	(1.3881,1.4293)		10	(1.0950,1.2056)	
			8	(1.4293,1.6286)		11	(1.2056,1.3244)	
			9	(1.6286,1.8197)		12	(1.3244,1.4332)	
			10	(1.8197,1.9590)		13	(1.4332,1.5124)	
			11	(1.9590,2.0000)		14	(1.5124,1.6183)	
						15	(1.6183,1.7215)	
						16	(1.7215,1.8210)	
						17	(1.8210,1.9180)	
						18	(1.9180,2.0000)	

## Data Availability

Data sharing not applicable.
